# Multifocal sclerosing angiomatoid nodular transformation of the spleen: a case report and review of literature

**DOI:** 10.1186/s13000-015-0312-2

**Published:** 2015-07-11

**Authors:** Zhixin Cao, Qiangxiu Wang, Jiamei Li, Jiawen Xu, Jianfeng Li

**Affiliations:** Department of Otorhinolaryngology Head and Neck Surgery, Shandong Provincial Hospital Affiliated to Shandong University, Jinan, P.R. China; Shandong Provincial Key Laboratory of Otology, NO 4, Duanxing Road (West), HuaiYin District, Jinan, P.R. China; Department of Pathology, Shandong Provincial Hospital Affiliated to Shandong University, Jinan City, Shandong Prov China

**Keywords:** Sclerosing angiomatoid nodular transformation, Multiple nodules, Laparoscopic splenectomy, IgG4-related sclerosing disease

## Abstract

Sclerosing angiomatoid nodular transformation (SANT) is a relatively new entity in the spleen, which usually presents in the form of single nodule. Only 5 multifocal SANT cases have been reported in English literature. The present case is the first report of a 38-years-old male patient with SANT in the form of multiple nodules, who has been cured via laparoscope. In comparison to solitary SANT, multifocal SANT occurs more likely in males than females and association with malignant neoplasm has not been described yet. Multifocal SANT as well as solitary SANT show some relationships with IgG4-related sclerosing disease.

**Electronic supplementary material:**

The online version of this article (doi:10.1186/s13000-015-0312-2) contains supplementary material, which is available to authorized users.

## Background

Sclerosing angiomatoid nodular transformation (SANT) is one kind of very rare benign lesion in the spleen, which may present as ., Solitary type or Multifocal type SANT. To date, a total of 127 cases of SANT have been reported in the English literature, of which only 5were multifocal [1–3]. In this report, we present a new case of multifocal SANT that had been treated with laparoscopic splenectomy successfully. We hope this report will help to accumulate more experience for an accurate diagnosis and proper therapy of multifocal SANT.

## Case presentation

A 38-years-old man suffered from dull pain in left upper quadrant of abdomen in December 2011. The pain became sharp when the body turns right lateral flexion, without other symptoms such as nausea, vomiting, abdominal distention, diarrhea. The patient denied referring to the physician for any consultant and treatment further.

**Fig. 1:**
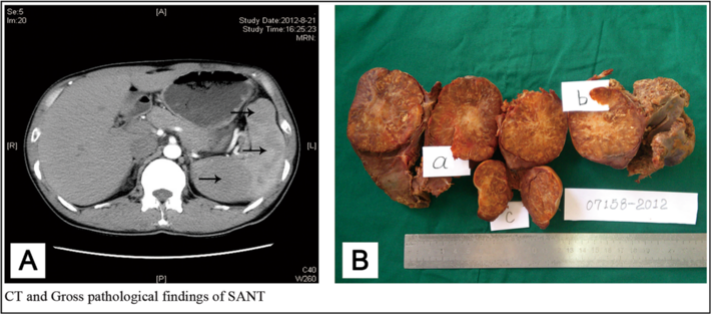
**a** Arterial phase scan showed marked multiple irregular nodular enhancement (arrows) with a few faintly visible stellar change (arrowheads) penetrating the center of the lesion from the periphery. **b** Three well- circumscribed round tumors were found on the cut surface measuring 6.5 cm (*a*), 5.0 cm (*b*) and 3.5 cm (*c*) in diameter respectively, the masses were non-encapsulated and grey brown and focal calcification with a grey white scar in the center of the lesion

On August 2012, abdominal CT scan was performed for the patient, which showed an enlarged spleen, with multifocal lobulated or irregular nodules in the spleen parenchyma. These nodules were isodense or hypodense, well-defined boundary, which were 3.5 cm, 5.5 cm and 7.6 cm in diameter respectively (Fig. 1a). The arterial phase and portal venous phase imaging both showed marked nodular enhancement with a few faintly visible stellate change penetrating the center of the lesion from the periphery. The delayed enhanced imaging showed that the lesion was progressively enhanced toward its center, becoming nearly isodense to the normal spleen. According to these imaging manifestations, the patient was initially diagnosed as multiple angioma, while metastatic neoplasm could not be completely excluded. Then, the patient was admitted to the department of hepatobiliary surgery in Provincial Hospital Affiliated to Shandong University for further therapy at August 22, 2012. The decision was made to proceed with an operation by laparoscopic splenectomy after the necessary laboratory investigations.

The patient change given laparoscopic splenectomy, and a new upper midline abdominal incision was made for removing the large spleen. The spleen was sectioned into pieces and placed in a specimen bag and were sent to Department of Pathology. The patient did well during the post-surgical period and discharged 14 days after the surgery without any complications.

**Fig. 2:**
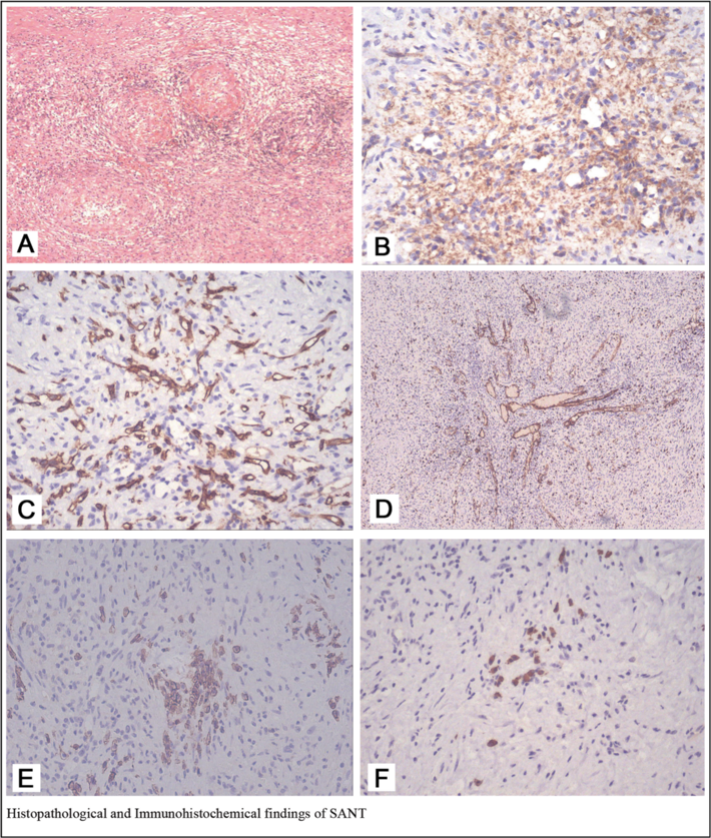
**a** Microscopically at low-power (×10), the lesion was composed of multiple, variably sized, circumscribed and confluent angiomatoid nodules with a central vascular core and a fibrosclerotic stroma on periphery. Different vessels in the nodules had distinct immunophenotypes, three kinds of vessels were positive for CD31 (**b**, × 40), the capillaries were positive for CD34 (**c**, ×40), and the dilated sinusoid like vessels were positive for CD8 (**d**, ×10). Plasma cells in the stroma revealed IgG+ (Fig. 2 **e**) and some few of which showed IgG4+ (Fig. 2 **f**) (<50/HP)

Macroscopically, the spleen was 18 cm × 11 cm × 8 cm in bulk volume, with three well-circumscribed round tumors on the cut surface which measured 6.5 cm, 5.0 cm and 3.5 cm in diameter, respectively, which was consistent with the impression of multifocal lesions on CT findings. Every mass was non-encapsulated and grey brown, focal calcification with a grey white stellate scar in the center of the lesion (Fig. 1b). Microscopically, at low-power, SANT was composed of multiple, variably sized, circumscribed and confluent angiomatoid nodules with a central vascular core and a fibrosclerotic stroma on periphery (Fig. 2a). On high power examination, three types of vascular structure were found in the tumor. One was capillary-like vessels, which were characterized by the presence of erythrocytes within the lumin, another was larger vessels, which resembled the sinusoids in the red pulp, and the third was ectatic veins, which usually had a well-defined vascular wall. Nuclear atypia was minimal, mitotic figures were extremely rare, and necrosis was not present. A variable number of plasma cells were present in the internodular sclerotic stroma and in the peripheral portion of the masses. Immunohistochemistry revealed that three different vessels in the nodules showed distinct immunophenotypes. The capillaries showed CD34+/CD8−/CD31+ and the ectatic small veins were CD34−/CD8−/CD31+, the dilated sinusoids like vessels presented CD34−/CD8+/CD31+. In other words, three kinds of vessels were all positive for CD31 (Fig. 2b), the capillaries were positive for CD34 (Fig. 2c), the dilated sinusoid like vessels were positive for CD8 (Fig. 2d). In the sclerotic stroma we can found some plasma cells which revealed IgG+ (Fig. 2e) and some of which showed IgG4+ (Fig. 2f) (<50/HP).

## Discussion

SANT of the spleen, as a new entity, was first described in detail in a series of 25 patients by Martel et al. [4] in 2004. Clinically, SANT is only a kind of described pathological diagnostic conception, the exact nature of this disease, however, is not fully understood. In this paper, we reported a case of multifocal SANT managed at our hospital and retrospectively reviewed medical records of 127 patients with SANT reported in English literature, including 97 cases reported by Falk et al. [5] in March 2012 and the additional 30 cases reported by other investigators dated from March 2012 to present(shows this in more detail in the Additional file 1: Table S1). In the present investigation, we retrieved a total of 128 cases (including this case) that comprised 57 males (44.5 %) and 71 females (55.5 %). The age ranged from 11–82 years old, with a mean age of 46 years, which was identical with the data reported by Falk and his colleagues [5]. Analysis showed that the ratio of male to female was 1:1.25, with a slight female predominance, which was basically consistent with the previous report (1:2) [5]. The present data, together with the other findings [6–15], suggested that the gender predilection may be prone to be neutralized as more cases were described, even though SANT had initially been considered to be a female predominant disease.

Of all 128 cases, 122 were solitary SANT, of which 53 were males and 69 were females; whereas, only 6 cases(include this case) were multifocal SANT, of which 4 were males and 2 were females, as shown in Table 1. Analysis showed that age of the 6 patients ranged from 31 to 57 years, with a mean age of 41 years and that the male–female ratio was 2:1, indicating a male predominance in comparison to the ratio of patients with solitary SANT (1:1.3). Evidently, as multifocal SANT is sparingly reported, the gender difference in these patients needs to be confirmed further with larger sample sizes.

**Table 1:** Clinical Features of 6 Cases of Multiple SANT of the Spleen

	Author	Age (year)	Gender	Spleen weight (g)	Gross features (size: cm)	Operation	Concurrent disease	Follow up
1	Diebole^1^ N = 1	56	Female	2400	3 nodules, 1.0, 2.0, 3.0 in diameter	splenectomy	Idiopathic myelofibrosis	NR
2	Kuo^2^ N = 3	39	Male	278	3 nodules, 1.2 × 0.9 × 0.8 to 0.9 × 0.8 × 0.7	splenectomy	Pancreatic mucinous cystadenoma	NED, 14 months
3		31	Male	654	8 nodules, 3.5 × 3 × 2.5 to 6.5 × 6.5 × 5	NR	Right inguinal mass	NED, 6 months
4		57	Male	142.5	3 nodules, 2 × 2 × 1.3, 2.4 × 1.9 × 1.5, and 4.8 × 4.5 × 4.4	NR	Left upper abdominal pain for 2 years	Lost follow up
5	J-C.Lee^3^ N = 1	43	Female	180	2 nodules, 3.5, 3.0 in diameter	splenectomy	Multiple calcifying fibrous pseudotumors	NR
6	Present case	38	Male	560	3 nodules, 3.5, 5.0, 6.5 in diameter	laparoscopic	hepatic cyst	NED, 26 months

Abbreviations: NED, No Evidence of Disease; NR, No Record

In this work, review of available documents in English literature, 30 of these 127 cases (23.6 %) coexisted with other diseases, such as idiopathic myelofibrosis, bile duct cancer, pancreatic cancer, acute pyelonephritis, and so on, most of which were cases of malignancy. Those results were matched up with Martel’s report [4] further confirming that patients with SANT, indeed, have a relatively high prevalence of coexistence with diseases at other organs. Such a situation reminds clinicians and radiologists that, once a splenic lesion is discovered and, particularly, coexists synchronously with malignant tumors at other sizes (for which metastatic tumors can not be ruled out, the possibility of patient with SANT should be considered integrating with imaging findings. We deem it advisable to do the necessary examination before or during operation to determine the explicit nature of the lesion. This can lead to avoid losing the best treatment opportunity due to the thinking of a tumor that has been widely metastasized. Feasibly, the needle aspiration or biopsy [16, 17] should be applied to clarify the nature of the space-occupying lesions in the spleen so as to select the optimal therapeutic regimen for achieving maximum efficiency.

Of 6 cases of multifocal SANT, 4 cases coexisted with other diseases, such as idiopathic myelofibrosis, multifocal calcifying fibrous pseudotumors, pancreatic mucinous cystadenoma and hepatic cyst, as shown in Table 1. Interestingly, none of these 6 cases coexisted with malignant tumors. Whereas, of 122 cases of solitary SANT, 26 cases (21.3 %) coexisted with other diseases, of which 15 cases (12.3 %) coexisted with malignant tumors. The prevalence of coexistence of multifocal SANT with diseases at other organs was higher than that of the solitary ones (0.01 < p < 0.05), in contrast, the tendency of coexistence of multifocal SANT with malignant tumors was likely lower than that of solitary ones. This phenomenon may be associated with that the cases of multifocal SANT are less, or that single and multifocal SANT, indeed, exist differences in terms of pathogenesis. This is necessary to accumulate more cases for the further textual research.

Macroscopically, the 5 reported patients with multifocal SANT presented multiple nodules (2–8 nodules), ranging in diameter from 1 to 6.5 cm. The characteristics of the present case were similar to those 5 patients. In addition to the differences in typical characteristic of multiple nodules and the clinical features mentioned above, the traits in terms of imaging [18], pathology and immunophenotype had no other differences from the solitary SANT [1–3]. As primary or metastatic tumors in the spleen are rare, and multiple space-occupying lesions in the spleen are extremely rare, thus, we should realize that SANT of the spleen can present in the form of multiple nodules.

Meanwhile, we found that a variable number of plasma cells were present in the internodular sclerotic stroma and a few of which showed IgG4 positive with immunohistochemistry. On average, the number of IgG4+ and IgG+ cells were 22/HPF and 95/HPF, respectively. The IgG4/IgG ratio was 23.1 %. Kuo et al. [2] reported three cases of multiple nodulars SANT and of which the mean IgG4 + cells/HPF and IgG4/IgG ratio were 7.7, 30.7, 52 and 9.5 %, 14.4 %, 19.7 % respectively. Analysis of the data of this case those reported by Kuo et al. [2] in 2009 revealed that the differences in the number of IgG4+ plasma cells between multifocal SANT and solitary SANT was not statistically significant (*P* = 0.705), but they were statistically significant (*P* = 0.009) with the differences between multifocal SANT and control spleens. Compared with normal spleens, the data showed some relationships between multiple SANT and IgG4-related sclerosing disease as well as solitary SANT, which presented evidence to support the hypothesis that this disease entity was associated with IgG4-related sclerosing disease as previous study reported by other literature [2, 8, 13].

SANT is a benign lesion, for which the curative treatment is splenectomy. Review of the existing literature revealed that only 8 cases of solitary SANT were treated with laparoscopic splenectomy. The treatment effect was similar to that by open operation according to the experience of follow up [5, 7, 10, 13, 14]. To the best of our knowledge, no laparoscopic splenectomy had previously been performed in patients with multifocal SANT.

## Conclusions

In summary, we reported a unique patient with multiple nodules of SANT of the spleen who underwent laparoscopic splenectomy successfully. In comparison to solitary SANT, multifocal SANT occurs more likely in males than females and incorporates with other diseases, in which malignant neoplasm has not been described yet. Multifocal SANT as well as solitary SANT show some relationships with IgG4-related sclerosing disease. When multiple lesions are found in spleen in the imaging examination, the clinicians and radiologists should be conscious of the possibility of multifocal SANT.

## Consent

Written informed consent was obtained from the patient for publication of this Case report and any accompanying images. A copy of the written consent is available for review by the Editor of this journal.

## Additional file

Additional file 1:
**Clinical Feature of 31 Cases of SANT By Other Investigators Dated from March 2012 to Present.**

## Abbreviations

SANT: Sclerosing angiomatoid nodular transformation
IgG4-RD: IgG4-related disease
CT: Contrast-enhanced computed tomography
HPF: High-power fields
