# Supplementary material for: Multifocal sclerosing angiomatoid nodular transformation of the spleen: a case report and review of literature
Source: Diagn Pathol. 2015 Jul 11;10:95. doi: 10.1186/s13000-015-0312-2 (PMC4498527; doi:10.1186/s13000-015-0312-2)
Supplement: Additional file 1: — Clinical Feature of 31 Cases of SANT By Other Investigators Dated from March 2012 to Present. [file 13000_2015_312_MOESM1_ESM.docx]

Additional Table

Clinical Feature of 31 Cases of SANT By Other Investigators Dated from March 2012 to Present

| **Author** | **Sex** | **Age(year)** | **Tumor size**  **(size: cm)** | **Surgery** | | **Together with other disease** | **Symptomatic** | **Follow-up** |
| --- | --- | --- | --- | --- | --- | --- | --- | --- |
| [Quirós JL](http://www.ncbi.nlm.nih.gov/pubmed?term=Quir%C3%B3s%20JL%5BAuthor%5D&cauthor=true&cauthor_uid=23596760)^[6](http://www.ncbi.nlm.nih.gov/pubmed?term=Quir%C3%B3s%20JL%5BAuthor%5D&cauthor=true&cauthor_uid=23596760)^  [N=1](http://www.ncbi.nlm.nih.gov/pubmed?term=Quir%C3%B3s%20JL%5BAuthor%5D&cauthor=true&cauthor_uid=23596760) | female | 68 | NR | operation | thrombocytopenia | | NR | NR |
| Budzyński A^7^  N=1 | female | 23 | 9 in diameter | underwent uneventful laparoscopic resection of the upper half of the splenic parenchyma | with 2-year history of recurrent mild fever, diffuse joint pain mostly of the small joints | | occasional vague abdominal discomfort in the middle abdomen | in long-term follow-up, improvement of preoperative symptoms and abnormalities in the blood tests was documented |
| [Nagai Y^8^](http://www.ncbi.nlm.nih.gov/pubmed?term=Nagai%20Y%5BAuthor%5D&cauthor=true&cauthor_uid=18752536)  N=3 | female | 46 | the spleen was 160g, and a whitish solitary nodule 3.3×3.0 in size | the spleen was resected after clinical diagnosis of metastatic tumour of unknown origin | NR | | an incidental splenic mass was detected on a general health examination | in the 4 years since the surgery, there has been no recurrence |
|  | male | 65 | the spleen was 140 g and a 1.0cm nodule | NR | cholangiocarcinoma | | NR | death bcause of cholangiocarcinoma. |
|  | female | 51 | the spleen was 210 g and a 5.5cm nodule | NR | NR | | an incidental splenic mass was detected on a general health examination | NED, 5 years |
| Watanabe M^9^  N=1 | female | 50 | 3.4×3.3×2.4 mass at the lower pole of the spleen | NR | NR | | found by ultrasonography during a health check-up  no abnormality | NR |
| WANG H L^10^  N=5 | man | 28 | 4.0 | laparoscopic | NR | | left upper abdomen pain | NED, 34 months |
|  | man | 52 | 2.0 | splenectomy | NR | | incidental finding | NED, 58 months |
|  | female | 40 | 7.0 | Open splenectomy | NR | | epigastric discomfort | NED, 60 months |
|  | female | 44 | 3.5 | Open splenectomy | NR | | vague abdominal pain | NED, 63 months |
|  | male | 46 | 5.0 | Open splenectomy | NR | | incidental finding | NED, 63 months |
| Raman SP^11^  N=5 | female | 37 | NR | splenectomy | NR | | incidentally discovered splenic mass while undergoing evaluation for a pancreatic lesion | NR |
|  | male | 67 | NR | splenectomy | NR | | incidentially discovered while undergoing for a pancreatic IPMN | NR |
|  | male | 49 | NR | splenectomy | ductal adenocarcinoma of the pancreatic head | |  | NR |
|  | female | 27 | NR | splenectomy | anemia | | incidentially discovered while undergoing CT examination for trauma | NR |
|  | male | 39 | NR | splenectomy | NR | | incidentially discovered while undergoing evaluation for possible ulcerative colitis | NR |
| Peter Kornprat^12^  N=1 | male | 69 | 5.8 in diameter. a slightly enlarged spleen with 320g | underwent surgery and a distal pancreatectomy with splenectomy | alcoholism, diabetes mellitus and arterial hypertension. | |  | NED, 16 months |
| Kim HH^13^  N=1 | female | 51 | 4 ×4 in size, hard multinodular mass | laparoscopic splenectomy | NR | | 1-week history of intermittent abdominal discomfort | NED, 2 months |
| [Agrawal M^14^](http://www.ncbi.nlm.nih.gov/pubmed?term=Agrawal%20M%5BAuthor%5D&cauthor=true&cauthor_uid=24715557)  N=2 | male | 35 | the spleen weighed 260g, cut surface showed well circumscribed mass measuring 6cm in diameter | NR | mildly elevated serum lactatedehydrogenase levels (124 U/L) | |  | NED, 11 months |
|  | girl | 12 | the mass had a nodular, firm to hard, gray white cut surface | splenectomy | NR | | with upper quadrant discomfort since 6 months | NED, 5 years |
|  | female | 52 | 4 ×5 hard multinodular mass in the perihilar upper pole area of the spleen | laparoscopic splenectomy | NR | | 2-week history of intermittent abdominal discomfort | NR |
|  | female | 53 | 4 ×6 hard multinodular mass in the perihilar upper pole area of the spleen | laparoscopic splenectomy | NR | | 3-week history of intermittent abdominal discomfort | NR |
|  | female | 54 | 4×7 hard multinodular mass in the perihilar upper pole area of the spleen | laparoscopic splenectomy | NR | | 4-week history of intermittent abdominal discomfort | NR |
| Kim HJ^15^  N=7 | female | 40 | Change from 1.9 to 2.7in diameter during the following-up period | No [surgical](http://cn.bing.com/dict/clientsearch?mkt=zh-CN&setLang=zh&form=BDVEHC&ClientVer=BDDTV3.5.0.4311&q=%E6%9C%AA%E8%BF%9B%E8%A1%8C%E6%89%8B%E6%9C%AF) intervention was [done](http://cn.bing.com/dict/clientsearch?mkt=zh-CN&setLang=zh&form=BDVEHC&ClientVer=BDDTV3.5.0.4311&q=%E6%9C%AA%E8%BF%9B%E8%A1%8C%E6%89%8B%E6%9C%AF) | cervical cancer | | NR | 12months |
|  | male | 39 | Change from 3.8 to 4.7in diameter during the following-up period | No [surgical](http://cn.bing.com/dict/clientsearch?mkt=zh-CN&setLang=zh&form=BDVEHC&ClientVer=BDDTV3.5.0.4311&q=%E6%9C%AA%E8%BF%9B%E8%A1%8C%E6%89%8B%E6%9C%AF) intervention was [done](http://cn.bing.com/dict/clientsearch?mkt=zh-CN&setLang=zh&form=BDVEHC&ClientVer=BDDTV3.5.0.4311&q=%E6%9C%AA%E8%BF%9B%E8%A1%8C%E6%89%8B%E6%9C%AF) | none | | NR | 16 months |
|  | female | 43 | 2.0 in diameter during the following-up period | No [surgical](http://cn.bing.com/dict/clientsearch?mkt=zh-CN&setLang=zh&form=BDVEHC&ClientVer=BDDTV3.5.0.4311&q=%E6%9C%AA%E8%BF%9B%E8%A1%8C%E6%89%8B%E6%9C%AF) intervention was [done](http://cn.bing.com/dict/clientsearch?mkt=zh-CN&setLang=zh&form=BDVEHC&ClientVer=BDDTV3.5.0.4311&q=%E6%9C%AA%E8%BF%9B%E8%A1%8C%E6%89%8B%E6%9C%AF) | none | | NR | 13 months |
|  | male | 42 | 3.9 in diameter | No [surgical](http://cn.bing.com/dict/clientsearch?mkt=zh-CN&setLang=zh&form=BDVEHC&ClientVer=BDDTV3.5.0.4311&q=%E6%9C%AA%E8%BF%9B%E8%A1%8C%E6%89%8B%E6%9C%AF) intervention was [done](http://cn.bing.com/dict/clientsearch?mkt=zh-CN&setLang=zh&form=BDVEHC&ClientVer=BDDTV3.5.0.4311&q=%E6%9C%AA%E8%BF%9B%E8%A1%8C%E6%89%8B%E6%9C%AF) | none | | NR | 0 months |
|  | male | 39 | Change from 4.0 to 4.5 in diameter during the following-up period | No [surgical](http://cn.bing.com/dict/clientsearch?mkt=zh-CN&setLang=zh&form=BDVEHC&ClientVer=BDDTV3.5.0.4311&q=%E6%9C%AA%E8%BF%9B%E8%A1%8C%E6%89%8B%E6%9C%AF) intervention was [done](http://cn.bing.com/dict/clientsearch?mkt=zh-CN&setLang=zh&form=BDVEHC&ClientVer=BDDTV3.5.0.4311&q=%E6%9C%AA%E8%BF%9B%E8%A1%8C%E6%89%8B%E6%9C%AF) | diabetes mellitus,hypertention | | NR | 8months |
|  | female | 48 | Change from 3.0 to 3.7in diameter during the following-up period | No [surgical](http://cn.bing.com/dict/clientsearch?mkt=zh-CN&setLang=zh&form=BDVEHC&ClientVer=BDDTV3.5.0.4311&q=%E6%9C%AA%E8%BF%9B%E8%A1%8C%E6%89%8B%E6%9C%AF) intervention was [done](http://cn.bing.com/dict/clientsearch?mkt=zh-CN&setLang=zh&form=BDVEHC&ClientVer=BDDTV3.5.0.4311&q=%E6%9C%AA%E8%BF%9B%E8%A1%8C%E6%89%8B%E6%9C%AF) | acute pyelonephritis | | Flank pain | 6months |
|  | male | 50 | 2.8 in diameter | No [surgical](http://cn.bing.com/dict/clientsearch?mkt=zh-CN&setLang=zh&form=BDVEHC&ClientVer=BDDTV3.5.0.4311&q=%E6%9C%AA%E8%BF%9B%E8%A1%8C%E6%89%8B%E6%9C%AF) intervention was [done](http://cn.bing.com/dict/clientsearch?mkt=zh-CN&setLang=zh&form=BDVEHC&ClientVer=BDDTV3.5.0.4311&q=%E6%9C%AA%E8%BF%9B%E8%A1%8C%E6%89%8B%E6%9C%AF) | early gastric cancer, GIST of jejunum | | NR | 0 months |
| Present case | male | 38 | 3 nodules with  6.5, 5.0 and 3.5 in diameter | laparoscopic  splenectomy | coexist hepatic cyst | | upper quadrant discomfort  since 6 months | NED, 22 months |

Abbreviations: NED, No Evidence of Disease; NR, No Record
